# Prioritization of Variants Detected by Next Generation Sequencing According to the Mutation Tolerance and Mutational Architecture of the Corresponding Genes

**DOI:** 10.3390/ijms19061584

**Published:** 2018-05-27

**Authors:** Iria Roca, Ana Fernández-Marmiesse, Sofía Gouveia, Marta Segovia, María L. Couce

**Affiliations:** Unit of Diagnosis and Treatment of Congenital Metabolic Diseases, Department of Pediatrics, Hospital Clínico Universitario de Santiago de Compostela, 15706 Santiago de Compostela, Spain; iria.roca.otero@gmail.com (I.R.); amarmiesse@gmail.com (A.F.-M.); sofiabsg@gmail.com (S.G.); m.segovia.amaro@gmail.com (M.S.)

**Keywords:** rare disease diagnosis, variant prioritization, mutation tolerance, mutational architecture, next-generation sequencing

## Abstract

The biggest challenge geneticists face when applying next-generation sequencing technology to the diagnosis of rare diseases is determining which rare variants, from the dozens or hundreds detected, are potentially implicated in the patient’s phenotype. Thus, variant prioritization is an essential step in the process of rare disease diagnosis. In addition to conducting the usual in-silico analyses to predict variant pathogenicity (based on nucleotide/amino-acid conservation and the differences between the physicochemical features of the amino-acid change), three important concepts should be borne in mind. The first is the “mutation tolerance” of the genes in which variants are located. This describes the susceptibility of a given gene to any functional mutation and depends on the strength of purifying selection acting against it. The second is the “mutational architecture” of each gene. This describes the type and location of mutations previously identified in the gene, and their association with different phenotypes or degrees of severity. The third is the mode of inheritance (inherited vs. de novo) of the variants detected. Here, we discuss the importance of each of these concepts for variant prioritization in the diagnosis of rare diseases. Using real data, we show how genes, rather than variants, can be prioritized by calculating a gene-specific mutation tolerance score. We also illustrate the influence of mutational architecture on variant prioritization using five paradigmatic examples. Finally, we discuss the importance of familial variant analysis as final step in variant prioritization.

## 1. Introduction

The growing use of next-generation sequencing (NGS) technology in large human populations has revealed that the extent of the variation in the human genome exceeds previous estimations. Moreover, it has been shown that the vast majority of protein-coding variation is evolutionarily recent, rare, and enriched for deleterious alleles, and that the rarest single nucleotide variants (SNVs) are population-specific [1]. Rare variation, therefore, likely contributes significantly to human phenotypic variation and disease susceptibility, particularly in the field of rare diseases.

When a rare disease diagnosis using NGS analysis (focused gene panel, whole exome sequencing (WES) or whole genome sequencing (WGS)) is requested, there is a high likelihood that the result will consist of a list of rare variants of uncertain significance. Many of these variants will prove benign. While most laboratories that perform NGS testing will provide an assessment of the pathogenicity of the variant, this should be interpreted with caution. Currently available informatics tools used to evaluate pathogenicity are limited to the assessment of nucleotide conservation throughout evolution and differences in the physicochemical features of the two amino acids involved in the change (in the case of missense variants) [2,3,4,5,6,7,8,9,10,11]. In the case of synonymous and intronic variants, developed software can determine whether the nucleotide change disrupts a consensus sequence that is particularly important for splicing (acceptor/donor sites, enhancer/silencing motifs, and branchpoints) [12,13]. Nonetheless, any rare variant has the potential to be pathogenic, irrespective of the predictions of bioinformatic tools. Conversely, many variants with predicted pathogenicity are often subsequently revealed to be benign. Whenever possible, sequencing of both parents and the proband will substantially increase the interpretability of any variants detected. However, when seeking a molecular diagnosis, it is essential to consider two additional levels of variant prioritization: the mutation tolerance and the mutational architecture of each gene, both of which are intimately related. With the aid of examples created using real data, this review will provide a comprehensive explanation of these two important concepts.

## 2. Mutation Tolerance

### 2.1. Introduction

To prioritize variants detected by NGS-based analysis, it is important to perform a quantitative assessment of the tolerance of each gene to functional genetic variation. Certain genes are much more tolerant to mutation than others. Experts who work with genes implicated in rare diseases are familiar with the variability of these genes in controls and patients, and understand that the appearance of rare variants in certain genes is not a cause for alarm, whereas in other cases, the appearance of one or (in the case of a recessive disease) two rare variants is highly indicative of pathogenicity.

It has long been known that the extent of conservation varies between genes [14]. Several studies of substitution-rate variation have shown that the local mutation rate varies across the mammalian genome. The substitution rate is affected by the proportion of GC (since the base composition is not equilibrated), the site itself (CpG or non-CpG), and the mutation type (e.g., transition, transversion, length variants) [15,16]. Petrovski et al. [17] developed a system to assign to each gene a score that measures intolerance to functional genetic variation, based on data extracted from 6503 whole-exome sequences from the NHLBI Exome Sequencing Project (ESP). They showed that genes responsible for Mendelian diseases are significantly less tolerant to functional genetic variation than genes that do not cause any known disease, and observed striking variation in intolerance among genes that cause different classes of genetic disease [17]. As mentioned above, many tools have been developed to attempt to prioritize variants in terms of measures of conservation at either the phylogenetic level or based on amino acid characteristics. However, few analogous approaches have been developed to prioritize the genes in which the variants are detected [18,19]. By quantifying the strength and consistency of purifying selection acting against functional variation in a given gene, or the effects of other kinds of selection (e.g., balancing or positive selection), genes can be ranked based on their mutation tolerance. Thus, a gene-level score could be combined with well-established variant-level scores to highlight candidate causal mutations.

### 2.2. Information That Can Be Extracted from Common and Rare Variants in Control Sample Databases to Evaluate Mutation Tolerance

For the more evolutionarily conserved genes (intolerant genes), which are subjected to negative-purifying selection, it is assumed that, for common variants, the non-synonymous mutation rate (Kn-syn) should be lower than the synonymous mutation rate (Ksyn), which involves greater a priori impact for new mutations. This is because if one variant is pathogenic, the probability that it will be perpetuated in the population in the form of common variant decreases dramatically as a consequence of the negative-purifying selection acting against it. By contrast, new mutations found in less conserved genes (tolerant genes) involve lesser a priori functional impact. For example, the analysis of data generated from the sequencing of 6500 “control” exomes as part of the ESP6500 project has been previously used to define the load of loss-of-function mutations per gene [20] and to prioritize >4000 human genes that are most intolerant to variation [21].

In the case of rare variants (the most important for rare disease diagnosis), in addition to gene conservation, we must bear in mind that the length and specific mutation rate of each gene will influence the number of variants observed per gene. This is because in the case of novel rare variants, not enough time has passed to observe the effect of conservation force. Thus we cannot tell whether over time a given variant will be allowed perpetuate (i.e., is neutral) or will be eliminated (because it is harmful). Therefore, in addition to knowing the specific mutation tolerance of each gene, it is important to quantify the number of rare variants in genes in population controls in order to have a more precise estimate of the mutational load of rare variants expected for each gene. Rare variant data obtained from controls can be used to estimate the variant load (and distribution) per gene.

### 2.3. Mutation Tolerance of Genes Involved in Metabolic/Neurologic Diseases: Examples

**Figure 1:**
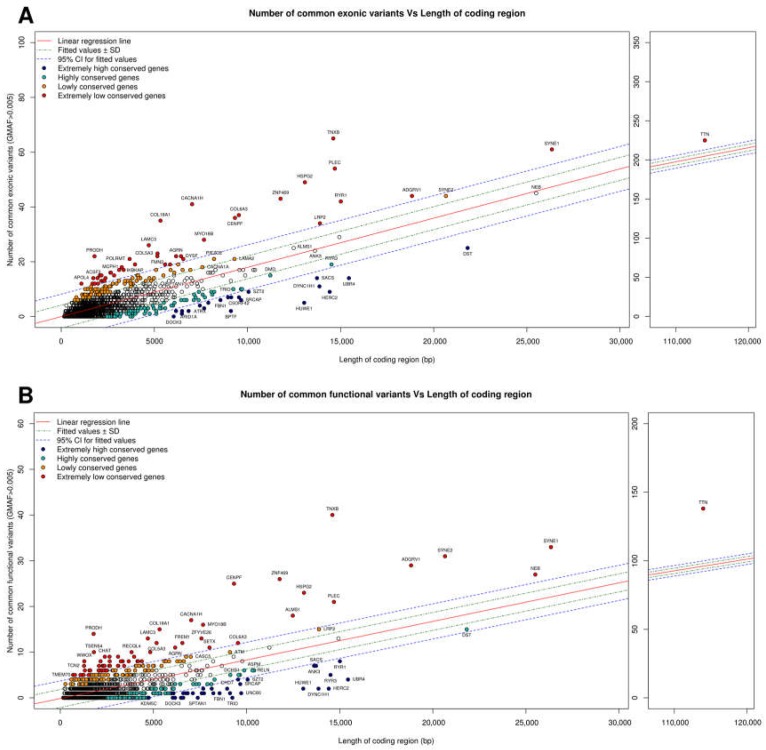

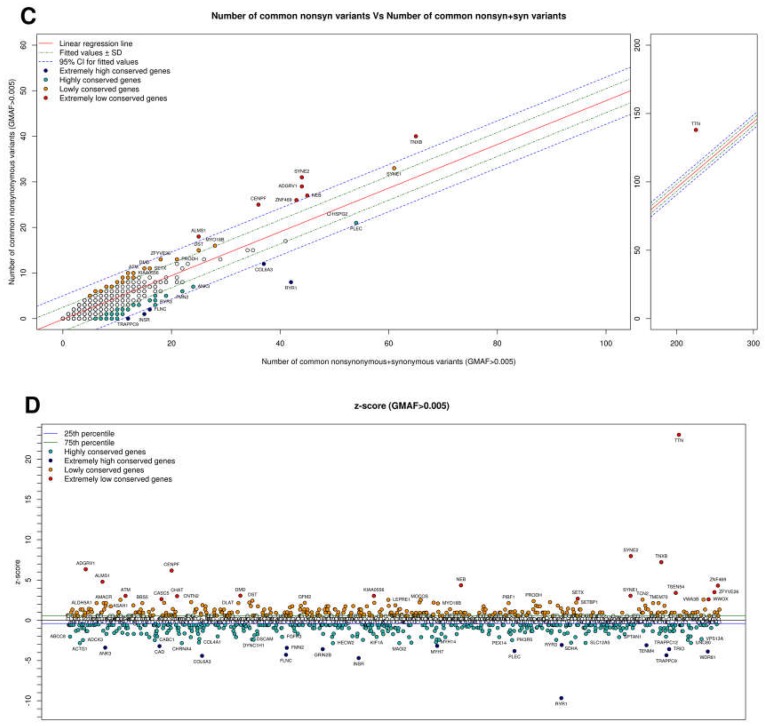
(**A**) Regression of number of common exonic variants (GMAF > 0.5%) against gene length for each of the 1670 genes analyzed; (**B**) Regression of number of common functional variants (all but synonymous, GMAF > 0.5%) against gene length for each gene; (**C**) Regression of number of non-synonymous common variants against number of non-synonymous and synonymous common variants (GMAF > 0.5%) for each gene; (**D**) *z*-score values for all analyzed genes.

To demonstrate the diversity of mutation tolerance among genes involved in rare disorders, we used the common variants detected in 659 samples from individuals of European ancestry extracted from the 1000 Genomes Project for each of the 1670 genes (additional data available upon request) included in our diagnostic panels for metabolic/neurologic rare diseases (www.neuromegen.com). We first regressed the number of total common exonic variants (minor allele frequency in 1000 G (GMAF) > 0.5%) against gene length (Figure 1A; *RSE* = 3.7, *AdjR*^2^ = 0.56). In this graph, the position of each gene is determined by two factors: (1) the specific mutation rate of the gene in question; and (2) the effect of conservation force on each gene (red genes exhibit more mutations than expected based on their size, and blue genes exhibit fewer variants than expected based on their size). Next, we plotted common functional variants (i.e., all except synonymous variants) against gene length (Figure 1B; *RSE* = 2.3, *AdjR*^2^ = 0.43). In this graph, more weight is given to evolutionary conservation force, since only variants that have functional consequences are considered. Compared with Figure 1, the position of most genes is unchanged, although in some cases, significant changes are observed, with some genes shifting position to the opposite side of the linear regression line. For example, the tolerance of *TTN*, *SYNE2*, *ADGRV1*, *CENPF*, *ALMS1*, *ZNF469*, and *NEB* is increased. While for some genes (e.g., *NEB*, *ALMS1*), the number of common variants is less than or equal to the number expected based on gene length, assessment of functional variants alone reveals a higher than expected mutational load, indicating that evolutionary conservation force does not exert a significant effect. Many of the genes for which this phenomenon was observed encode large structural proteins involved in muscular function (*TTN*, *NEB*, *SYNE2*). The opposite effect is observed for other genes (e.g., *RYR1*, *RYR3*, *ANK3*, *TRIO*, *SPTAN1*), for which the number of common variants is greater than that expected based on gene length (indicative of a high mutation rate for these genes). For these genes, restriction of the analysis to functional variants resulted in decreased mutation tolerance, with some genes (e.g., *RYR1*) shifting position to the other side of the linear regression line, indicating a significant effect of evolutionary conservation force on this gene (i.e., mutation intolerance). By regressing common non-synonymous variants against the total mutational load of the gene (common non-synonymous + synonymous) (Figure 1C; *RSE* = 1.3, *AdjR*^2^ = 0.81), we should discount the specific gene mutation rate for each gene and focus only on the weight of conservation force. This results in shifts in the positions of many genes from both the mutation-tolerant and mutation-intolerant sides of the graph to more neutral positions. Exceptions include *RYR1*, for which a decrease in tolerance is observed, and *COL6A3*, *PLEC*, and *HSPG2*, which shift from a mutation-tolerant to mutation-intolerant or neutral positions, indicating that their previous position was attributable only to their high specific mutation rate. For other genes, such as *TTN* or *ATM*, shifts in position reflecting increased mutation tolerance are observed, indicating that their previous positions were attributable to their a priori low mutation rate (e.g., *DST* moves from a mutation-intolerant to a mutation-tolerant position).

**Figure 2:**
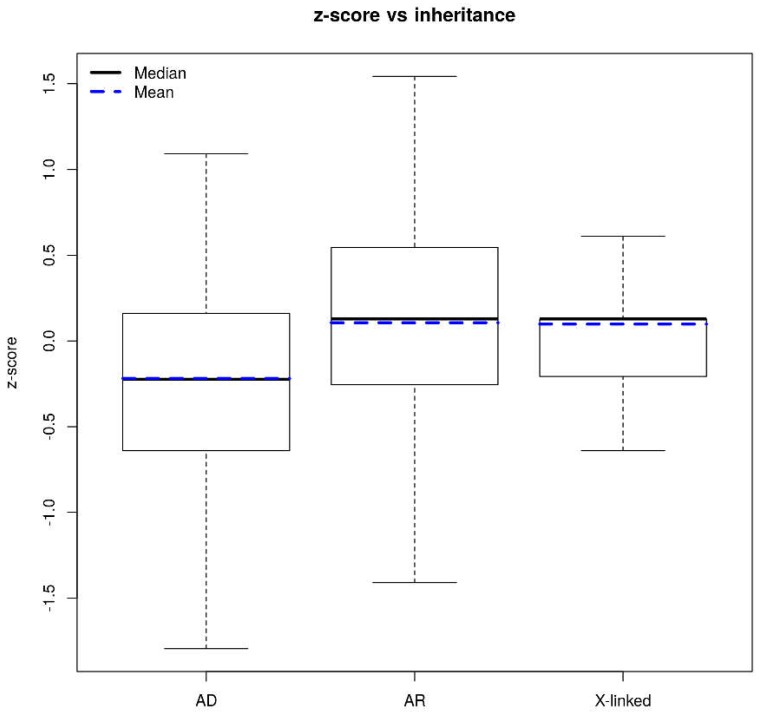
Boxplot (without outliers) of *z*-score values according to gene inheritance. AD: autosomal dominant; AR: autosomal recessive; X-linked.

We calculated the *z*-score for each gene to quantify mutation tolerance using a previously described method [17,21], which is based on departure from the mean common non-synonymous variant spectrum (functional), corrected for the total mutation burden in a given gene. The *z*-score was generated by regressing the total number of common (GMAF > 0.5%) non-synonymous variants against the total number of common variants (non-synonymous + synonymous) observed within a gene, after which each gene was assigned the corresponding studentized residual as its *z*-score. A *z*-score of 0 indicates that the number of non-synonymous variants in a given gene is equivalent to the number estimated based on its total mutation burden. Genes with a negative *z*-score have fewer than the expected number of common non-synonymous variants based on the global mutational burden, and are thus likely to be less tolerant of functional mutations. A negative *z*-score is therefore indicative of genes subjected to strong purifying selection. Figure 1D shows the *z*-score calculated for each of the 1670 genes from our diagnostic panels. The higher the *z*-score obtained for a gene, the more tolerant it is to functional mutations and the lower the probability of a priori pathogenicity. The lower the *z*-score of a given gene, the higher the a priori probability that a new mutation will be pathogenic or related to the phenotype, although there are exceptions to this rule depending on the gene length, as we will show later. As it could be expected, dominant genes have a much lower *z*-score than recessive genes (Wilcoxon test: *p*-value = 1.4 × 10^−13^) (Figure 2). Intuitively, dominant genes should be less tolerant to variation than recessive genes, since in the former case, perturbation of one allele can promote disease, while in the case of recessive genes, the second allele can compensate for the damaged allele. Purifying selection force is greater for dominant genes. We found that 50.4% of X-linked genes had the same *z*-score (equal to the median of all X-linked and recessive genes analyzed), which entails that 77% of X-linked genes have a *z*-score lower or equal to the median of recessive genes. This makes sense since the X-linked genes in males behave as dominant since they are reduced to hemizygosity, so they do not have a second allele that compensates for the damaged one.

The five genes more intolerant (lowest *z*-score) and tolerant to mutation (highest *z*-score) for each of the lengths tracts are presented in Table 1.

The genes listed in the “most tolerant” column (right side of Table 1) are, in fact, genes that appear very often in the final dataset of filtered rare variants produced by our diagnostic panels. Moreover, most of the mutations in these genes which had been previously associated with pathologies are truncating mutations, indicating that these genes are more permissive to missense mutations. The most tolerant gene by far is *TTN* (*z*-score: 24). The mean number of rare missense *TTN* variants (with GMAF < 0.5%) found in any individual is 0.5. The 364-exon *TTN* gene (OMIM 188840) encodes titin, the largest known human protein [22]. Titin is one of the most important sarcomeric proteins, and is involved in the development of cardiac and skeletal muscle, as well as in architecture, elasticity, and cell signaling. *TTN* mutations are associated with a broad spectrum of phenotypic severity [23,24,25,26,27,28,29,30]. In our experience, the detection of rare missense variants in the *TTN* gene in patients with neuromuscular disorders is relatively common, and does not necessarily indicate the involvement of this gene in the patient’s phenotype. In fact, most of the *TTN* variants recorded in ClinVar that are implicated in titinopathies are truncating variants. Only some missense mutations in trans with a truncating variant or isolated missense mutations located in highly specific protein codons have been associated with diseases [23].

Mutation-intolerant genes (left side of Table 1) are not usually observed in the final dataset of filtered rare variants produced by our diagnostic panels. When these genes do appear, we subject them to careful analysis to evaluate the compatibility between the associated disease and the patient’s clinical history, since the genes will likely be implicated in the patient’s disorder. However, as described below, the length of the gene should also be considered in this evaluation.

### 2.4. Information Extracted from Rare Variant Spectrum for Genes Implicated in Metabolic/Neurologic Diseases: Examples

**Figure 3:**
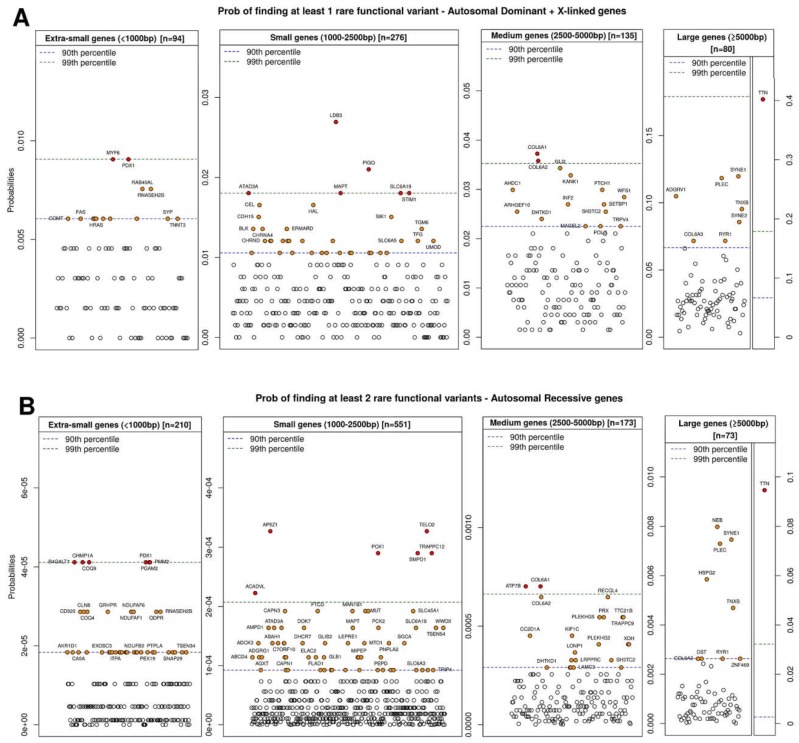
(**A**) Probability of finding at least one rare functional variant (all but synonymous, GMAF < 0.5%) in autosomal dominant and X-linked genes, grouped by gene length: extra-small genes (<1000 bp), small genes (1000–2500 bp), medium genes (2500–5000 bp), and large genes (>5000 bp). (**B**) Probability of finding at least two rare functional variants (all but synonymous, GMAF < 0.5%) in autosomal recessive genes, grouped by gene length.

The presence of rare variants in a gene not only depends on the specific mutation tolerance of the gene in question (previous section), but is also influenced by the gene’s length and its specific mutation rate. To illustrate this, using data from the same 659 probands analyzed in the previous section, we calculated the probability of finding one rare variant in dominant or X-linked genes or two rare variants in recessive genes for each of the 1670 genes analyzed (Figure 3). Genes that behaved as both dominant and recessive were included in both groups. To this end, we used the Poisson distribution, *P*(λ), where λ is the frequency of rare variants (GMAF < 0.5%) in each gene. Since gene length influences this probability, we also grouped genes based on length. As expected, *y*-axis values increased with increasing gene length for both groups (Figure 3A,B). It is not unusual to find two or more functional rare variants in recessive genes with high *z*-scores (i.e., mutation-tolerant genes), exemplified in this case by *TTN* (9%) and *NEB* (0.8%). Nonetheless, for several genes with low *z*-scores (intolerant), including *RYR1*, *RYR3*, *COL6A3*, and *PLEC*, the probabilities are in the same range (0.3%, 0.2%, 0.3% and 0.7%, respectively), most likely due to their large gene length.

The position occupied by a given gene in these graphs is of vital importance for the prioritization of rare variants detected by NGS analysis. Table 2 shows the dominant (left) and recessive (right) genes for which the probability of finding one or two variants, respectively, by chance, was lowest.

As shown in Table 2, a large percentage of genes for which the lowest probabilities were obtained, especially recessive genes, are implicated in basic human metabolism (red). Furthermore, a large percentage of the dominant genes for which the probability of finding one variant (usually a de novo mutation) was lowest are implicated in epileptic encephalopathy and brain development (green).

In the case of recessive genes, the presence of 2 rare variants (or a homozygous rare variant) in *GM2A*, *NEU1*, *DDHD1*, and *ARFGEF2* for example (in which the probability of detection by chance ranges from ~0% to 0.001%), dramatically increases the probability that these changes are associated with the patient’s phenotype, even if the patients’ clinical features do not match perfectly with the disease-associated gene. The opposite occurs for *TTN*, *NEB*, and *SYNE1*, for which the probability of finding two rare variants by chance is 9%, 0.8%, and 0.7%, respectively. However, it is important to note that the probability is based not only on the conservation score of each gene but also on gene length. Thus, despite the low *z*-scores of *RYR1*, *RYR3*, and *PLEC*, probabilities of 0.3%, 0.2%, and 0.7% were obtained, attributable to the length of the genes and possibly the effects of other factors influencing the mutation rate.

In the case of dominant genes, the detection of one rare variant in *STX1B*, *GABRG2*, *TUBB2B*, *TUBB*, *TUBB1A*, *GRIN2D*, *CHD2*, *SCN2A*, and *SCN1A*, all of which are implicated in epileptic encephalopathies or brain development and have scores ranging from ~0% to 1%, warrants careful attention and indicates a need to perform familial studies, since these mutations are likely de novo.

### 2.5. Conservation Score for Nucleotide(s) Affected in the Variant and Relationship with Variant Frequency

Once mutation tolerance and rare mutational load are assessed for each gene, it is important also to take into account a score that considers whether the specific nucleotide affected by the variant exhibits evidence of selective constraint throughout evolution. While a given gene may be highly mutation-intolerant, if the specific nucleotide that is mutated is not conserved, it is possible that the variant is neutral and vice versa (i.e., in mutation-tolerant genes, if the specific nucleotide is highly conserved, it is possible that the variant is harmful). There are bioinformatics tools that can quantitatively estimate nucleotide conservation throughout evolution [2,3].

**Figure 4:**
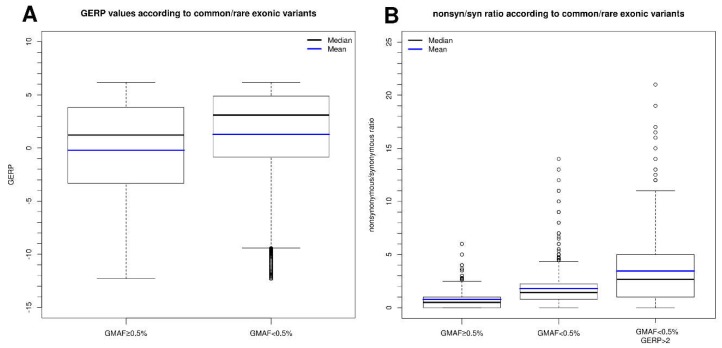
(**A**) Boxplot of GERP (Genomic Evolutionary Rate Profiling) values according to common (GMAF > 0.5%) and rare (GMAF < 0.5%) exonic variants. (**B**) Boxplot of non-synonymous/synonymous ratio values of each gene according to: common (GMAF > 0.5%), rare (GMAF < 0.5%), and highly conserved rare variants (GMAF < 0.5%, GERP > 2).

The GERP (Genomic Evolutionary Rate Profiling) tool assigns a specific conservation score to each position using maximum likelihood evolutionary rate estimation. As previously demonstrated [14], the data obtained with our group of genes indicate that the higher the GERP value, the lower the variant frequency (Figure 4A), and show that the non-synonymous-to-synonymous ratio among rare (<0.5%) variants (with GERP > 2) is higher (typically in the range of 1–2) than that observed for common variants (range, 0.5–1.5). This suggests that 25–50% of rare non-synonymous variants are deleterious (and therefore subjected to purifying selection) (Figure 4B). Based on these findings, we can infer that the lower the GMAF and the higher the GERP score, the greater the probability that the variant in question is implicated in a pathogenic process. This also implies that when we find, for example, a synonymous or intronic mutation that is not described in any population database (frequency = 0) and has a high GERP score (>4), it is imperative to analyze this change using a program to evaluate regions involved in splicing, since the degree of conservation should indicate whether the nucleotide in question is important for the functionality of the gene studied.

## 3. Mutational Architecture

After determining how tolerant a given gene is to mutation, the next step in the variant prioritization process is to assess, for a given tolerance score, the types of mutations and their position within the gene, and the association between these variants and different phenotypes. This is known as mutational architecture. It is well known that perturbation of different functional regions of one gene can affect different functions and processes, giving rise to distinct phenotypes [31]. Furthermore, different types of mutation (truncating vs. missense, gain vs. loss of function) can give rise to distinct phenotypes. This is more important in the case of rare disorders; recent research findings have demonstrated a marked overlap between phenotypic spectrums arising from mutations on different genes. Knowledge of the specific mutational architecture of each gene allows us to prioritize variants detected by NGS analysis. Assessment of the possible involvement of a variant in a given phenotype must consider the specific mutational architecture of the gene in which the mutation is situated. The same variant (type and location) could have very deleterious effects in one gene, but not in another. Certain genes are susceptible only to truncating variants but tolerate missense variants (e.g., *TTN*, *SYNE1*). Similarly, some genes (e.g., *KCNQ2*) are only susceptible to missense gain-of-function mutations, while truncating mutations give rise to much milder phenotypes. *SETBP1* is an example of a gene that is sensitive only to missense variants that are located in very specific positions of the protein and give rise to a very severe phenotype. In the case of *CDKL5*, specific exons of the gene are mutation-insensitive, while in some genes that only tolerate mosaic mutations, such as *AKT1* and *GNAS*, germinal mutations are lethal. Having up-to-date information on the specific mutational architecture of each gene allows correct prioritization of variants detected by NGS analysis, as illustrated below in 5 paradigmatic examples of mutations implicated in neurologic and metabolic disorders. In each example, specific aspects of the mutation must be taken into account to ensure correct variant prioritization.

### 3.1. KCNQ2: Disease Severity Depends on the Mutation Type (Truncating vs. Missense)

In some genes, different mutation types (truncating vs. missense) result in different degrees of pathological severity. Contrary to expectation, truncating mutations do not always give rise to the most severe phenotype, which depends on the function of the protein encoded by the gene. This is well exemplified by the *KCNQ2* gene. Mutations in this gene, which encodes one of the two subunits of the voltage-gated potassium channel that mediates the neuronal M-current (IKM) [32], give rise to early-onset epileptic diseases, which vary considerably in their phenotypic presentation [33]. The M channel mediates the conduction of a slowly activating and deactivating potassium current, and plays a major role in setting the lower electroexcitability threshold, as well as the capacity of neurons to respond to synaptic input. In 1998, the first dominant mutations in this channel were described, and were associated with benign familial neonatal epilepsy (BFNS) with good prognosis [33,34,35]. In stark clinical contrast, *KCNQ2* mutations were subsequently implicated with early onset epileptic encephalopathy (EOEE), a much more severe and disabling disorder [36]. Patients with *KCNQ2* encephalopathy display tonic seizures two to three days after birth, have an electroencephalography profile characterized by suppression-burst patterns or multifocal epileptic activity, and have a poor neurological prognosis [37,38,39]. The presence of *KCNQ2* whole-gene deletions in BFNS patients demonstrates that total loss of function of one allele causes only benign phenotypes. Therefore, haploinsufficiency cannot explain the more severe *KCNQ2*–EOEE phenotype. Moreover, all *KCNQ2*–EOEE mutations described to date are missense mutations, never haploinsufficiency mutations (which are always associated with BFNS). A possible explanation for this finding is that haploinsufficiency may be physiologically reversed, as observed in BFNS patients, while alleles with missense mutations can give rise to M channels that are functional but have varying voltage sensitivities depending on the amino acid change in question (gain and loss of function). This finding is very important since it could lead to the development of personalized treatments for *KCNQ2*–EOEE. Therapies using antisense oligonucleotides are being developed for diseases caused by known dominant negative mutations. Specific inhibition of the transcription or translation of the mutated *KCNQ2* allele could result in a loss-of-function scenario, mimicking *KCNQ2* haploinsufficiency, which in turn is known to give rise to the milder BFNS phenotype. Such a strategy could therefore potentially convert severe EOEE to a benign neonatal epilepsy syndrome [40].

### 3.2. SETBP1: Phenotypes Differ Markedly Depending on the Type of Mutation, the Location Thereof, and the Percentage of Cells Affected

*SETBP1* provides a particularly interesting example since the phenotype produced by mutations in this gene depends not only on the type of mutation (truncating vs. missense) but also on the region in which the missense mutation is located and the percentage of cells in which it is found (mosaic vs. germline mutations).

Haploinsufficiency mutations in this gene result in intellectual disability (ID) of variable severity associated with difficulties in verbal communication [41,42]. Although the spectrum of intellectual impairment ranges from normal to severe, mild ID is observed in most cases. Several individuals with disruptive mutations have good receptive language skills but completely lack expressive speech [20,43,44]. Other features include impaired fine motor skills, subtle dysmorphism, hyperactivity, and autistic traits [20,42].

**Figure 5:**
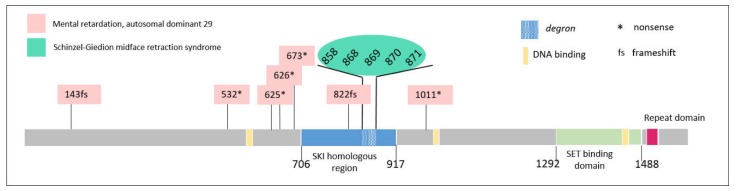
Structure of *SETBP1* and sites of pathogenic mutations associated with Schinzel-Giedion syndrome and mental retardation.

However, missense de novo mutations in three specific amino acids clustered in a highly conserved 11-bp exonic region (Figure 5) that includes residues 868 to 871 result in a very severe disorder known as Schinzel–Giedion syndrome (SGS), the first dominant disorder for which the genetic basis was identified by whole-exome sequencing [45]. It is characterized by severe mental retardation, persistent feeding problems, severe forms of epilepsy, distinctive facial features, and various congenital organ defects, blindness, deafness, and increased risk of malignancy. Most affected individuals die before the age of 10.

The same mutations were reported in somatic mosaicism in several types of myeloid malignancies after the association of de novo germline mutations in *SETBP1* with this syndrome [46,47,48]. This finding is unsurprising, since several other genes have been implicated in both developmental disorders and cancer, including *HRAS* (Costello syndrome), *ASXL1* (Bohring-Opitz syndrome), *EZH2* (Weaver syndrome), and *FGFR2* (Pfeiffer, LADD, Jackson-Weiss, Crouzon, Saethre-Chotzen, Apert, Beare-Stevenson cutis gyrata, bent bone dysplasia, and Antley-Bixler syndromes) [49]. These findings partially explain the high rates of childhood cancer in patients with congenital developmental defects [50,51,52], given that common molecular pathways may mediate both embryogenesis and cancer development [53,54].

Mutations in this *SETBP1* hotspot disrupt a degron, a signal that regulates protein degradation, leading to the accumulation of SET-binding protein 1. Interestingly, differences in phenotype severity depending on the amino acid changed in the degron sequence have also been reported [55]. The stability and protein levels of *SETBP1* are affected differently depending on the type of mutation, both within and around the degron, as has been demonstrated in both in vitro and by in silico modeling. Changes in residue I871 result in mild increases in protein expression, and are found at a significantly greater frequency in SGS versus leukemia. On the other hand, substitutions in residue D868 result in the greatest increases in protein levels, and are associated with a higher cell proliferation rate in vitro and a greater incidence of cancer as compared with patients with other germline *SETBP1* mutations. The degron is therefore more affected by somatic *SETBP1* mutations that promote cancer than by germline mutations that cause SGS, implying that the threshold for cancer caused by mutations in the *SETBP1* degron is higher than that for prenatal developmental alterations in SGS.

### 3.3. TCF4: Phenotype Depends on the Longitudinal Location of the Mutation in the Gene

**Figure 6:**
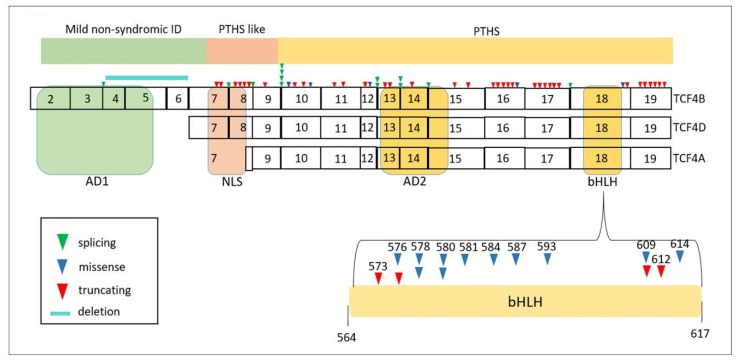
Structure of *TCF4* and locations of pathogenic mutations registered in ClinVar. AD1, first activation domain; AD2, second activation domain (AD1 and AD2 modulate transcriptional activity); NLS, nuclear localization signal (controls subcellular localization); bHLH, basic helix-loop-helix.

Phenotypes produced by variants in *TCF4* range from mild cognitive impairment to Pitt-Hopkins syndrome (PTHS), a neurodevelopmental disorder characterized by ID and characteristic facial features, in some cases accompanied by respiratory abnormalities, microcephaly, ophthalmological problems, epilepsy, or behavioural problems (autism spectrum disorder) [56,57,58,59,60,61]. The human *TCF4* gene coded by 18 exons and the last 3′ UTR exon is predicted to encode at least 18 different proteins with distinct N-terminal sequences. *TCF4* possesses numerous 5′ initial exons, and can thus generate a variety of different transcripts, all of which share the same final 12 exons (11 protein-coding exons and the last 3′ UTR exon) [62,63] (Figure 6). Depending on the location of the variants along the length of the *TCF4* gene (from 5′ to 3′), one or more transcripts of the gene, and the corresponding protein isoforms, will be affected. This gives rise to a variety of phenotypic presentations along the same clinical spectrum. Impairment of different protein domains of TCF4 protein gives rise to a variable clinical presentation [61] (Figure 6). Variants in exons 1–6 result in mental retardation that is mild but does not fall within the spectrum of Pitt–Hopkins syndrome [64,65,66,57]. These types of variants affect the longer transcript, but spare many others transcripts that are initiated later, in which both NLS and bHLH are conserved in both alleles. Variants in exons 7 and 8 result in impairment limited to the NLS domain in several transcripts and cause a more severe phenotype that resembles Pitt–Hopkins syndrome (PTHS-like), albeit without the characteristic facial dysmorphism. This is because they allow the existence of shorter transcripts with an intact bHLH domain. Variants in exons 9–18 give rise to the most severe Pitt–Hopkins syndrome phenotype, since all isoforms are affected and both essential domains NLS and bHLH are reduced to haploinsufficiency.

Interestingly, while most disease-associated variants in TCF4 are truncating mutations, more missense than truncating mutations are found in exon 18, which encodes the bHLH domain (Figure 6). The N-terminal region of the bHLH domain contains a highly conserved group of positively charged amino acids that directly interacts with DNA. The remainder of the bHLH motif forms a surface that favors homodimerization and heterodimerization through the assembly of as stable 4-helix bundle with a defined hydrophobic core.

### 3.4. LMNA: The Malleable Gene Paradigm

In this gene, mutations of different types in different locations, influenced by genetic background and/or epigenetic and environmental factors, give rise to very different and apparently unrelated phenotypes.

*LMNA* encodes lamins A and C, which are produced in roughly equal amounts. Lamins A and C are structural proteins located in the nuclear lamina, a fibrillar network consisting of intermediate filaments and other proteins that adheres to the inner membrane of the nuclear envelope, which regulates molecular transport in and out of the nucleus. Lamins A and C, which are also found inside the nucleus, may influence the expression of specific genes. Before being incorporated into the lamina, lamin A must undergo processing within the cell. Its precursor, prelamin A, undergoes a complex series of steps that are required to enable insertion of the protein into the lamina layer. This preprocessing stage is not required for incorporation of lamin C into the nuclear lamina.

**Figure 7:**
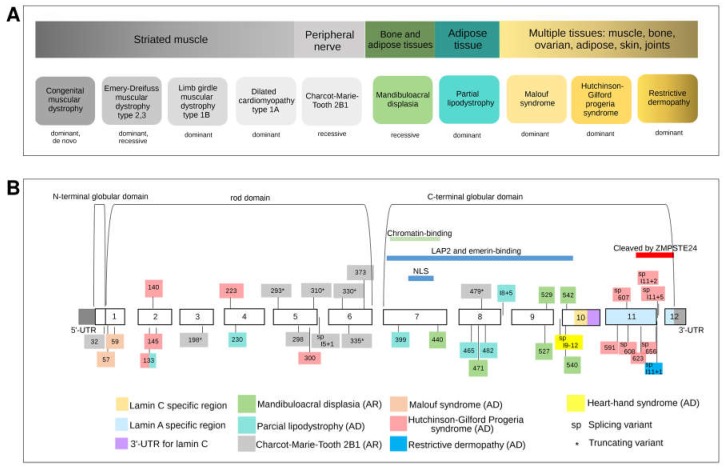
(**A**) Phenotypic spectrum of laminopathies and the different tissues affected. (**B**) Location of pathogenic mutations associated with different *LMNA* phenotypes.

Mutations in *LMNA*, depending on their type and location, may alter different lamin A/C functions and affect various processes, leading to a spectrum of genetic disorders, collectively referred to as laminopathies. *LMNA* mutations can affect skeletal and cardiac muscle, fat storage and metabolism, cell differentiation, DNA replication, and gene regulation. Hundreds of *LMNA* mutations (193 of which classified as pathogenic in ClinVar) have been described in patients. Mutations in *LMNA* have been implicated in about a dozen clinical disorders since the end of the 20th century. These can be classified as diseases that predominantly affect (1) striated muscle, (2) adipose tissue, (3) peripheral nerves, or (4) multiple tissues (Figure 7A). Lamin C, which is widely expressed in most differentiated somatic cells and some undifferentiated cells, but not in early embryos, is encoded by exons 1–9 and part of exon 10 (Figure 7B). Lamin A is the result of alternative splicing, which adds exons 11 and 12 and removes the lamin C-specific portion from exon 10.

#### 3.4.1. Striated Muscle Diseases

Numerous mutations located throughout the entire *LMNA* gene have been implicated in autosomal dominant, autosomal recessive, and sporadic cases of Emery-Dreifuss muscular dystrophy 2 and 3 [67,68,69]. Patients with this disorder develop early contractures of the elbows, Achilles tendons, and neck, spinal rigidity, muscle weakness that affects the upper arms and lower legs and worsens over time, and dilated cardiomyopathy with atrioventricular conduction block of early onset. Most of these cases are caused by missense mutations in exons that encode the central rod domain common to both lamins A and C. These mutations occur at evolutionarily conserved residues that likely have essential structural or functional roles. The resulting protein misfolding or assembly failure leads to partial or complete loss of function [70]. *LMNA* mutations have also been implicated in other myopathies, including limb girdle muscular dystrophy type 1B (LGMD1B) [71,72,73] and dilated cardiomyopathy type 1A (DCM1A) [74].

In dilated cardiomyopathy 1A, little or no skeletal muscle involvement is observed, while limb-girdle muscular dystrophy skeletal muscle around the shoulders and hips is predominantly affected, with sparing of the distal extremities and an absence of the joint contractures typical of classical Emery-Dreifuss muscular dystrophy. It was originally proposed that Emery-Dreifuss muscular dystrophy, limb girdle muscular dystrophy type 1B, and dilated cardiomyopathy 1A resulted from distinct *LMNA* mutations. However, environmental factors or the influence of other genes are more plausible explanations for this phenotypic variability. These three disorders (with overlapping phenotypes) could be considered different manifestations of the same disease. This hypothesis is supported by the results of a study [75] in which different members of the same family displayed the three distinct muscular phenotypes caused by a single mutation, suggesting that the phenotype is influenced by genetic background as well as environmental and epigenetic factors. Although most *LMNA* mutations that cause muscle disorders present during childhood or early adulthood, in rare cases patients present with congenital muscular dystrophy [76] of an earlier onset and more severe phenotype. While most cases of *LMNA*-associated congenital muscular dystrophy are caused by de novo mutations, cases caused by germinal mosaicism have been described [77].

#### 3.4.2. Heart-Hand-Foot Involvement

In 2008, heart-hand syndrome of Slovenian type was described as an adult-onset progressive sinoatrial and atrioventricular conduction disease, which results in dilated cardiomyopathy, a peculiar form of brachydactyly (mild hand involvement and more severe foot involvement), and sudden death caused by ventricular tachyarrhythmia. Alterations of the hands include short distal, middle, and proximal phalanges and clinodactyly, while foot alterations include short distal and proximal phalanges and metatarsal bones, short or absent middle phalanges, extra ossicles, syndactyly, terminal symphalangism, and duplication of the bases of the second metatarsals [78]. One *LMNA* mutation has been linked to this phenotype.

#### 3.4.3. Adipose Tissue Involvement

*LMNA* mutations also cause defects that affect fat storage and metabolism. Lipodystrophies are characterized by complete or partial loss of subcutaneous fat. Patients with Dunnigan-type familial partial lipodystrophy, a dominantly inherited disorder, do not exhibit any alterations of the subcutaneous adipose tissue until puberty, when they experience a regional loss of fat from the extremities associated with insulin-resistant diabetes mellitus with acanthosis nigricans and hypertriglyceridemia [76]. In some cases, the accumulation of subcutaneous fat on the face and neck can lead to a moon-shaped face, as well as a double chin or fat neck, in affected individuals. This phenotype is due almost exclusively to autosomal dominant missense mutations in exons 7–10 of *LMNA* [79,80,81,82] (Figure 7B). These mutations lie in the C-terminal globular tail region and may alter its interactions with protein targets [83,84]. At the cellular level, some mutations have been shown to cause structural defects in the nuclear envelope, fragile nuclei, and chromatin detachment from the lamina [82].

#### 3.4.4. Peripheral Nerve Involvement

By 2000, it was clear that both dilated cardiomyopathy and partial lipodystrophy were caused by *LMNA* mutations. However, the situation was further complicated when just a few years later, a homozygous amino-acid substitution (R298C) in this gene was linked to Charcot-Marie-Tooth disease type 2 [85]. This mutation lies in the conserved rod domain and is predicted to interfere with lamin binding. Again, phenotypic variability was demonstrated, indicating the influence of genetic/epigenetic factors in the resulting phenotype [86]. This finding suggested that nerve degeneration could account for the muscle wasting and degeneration observed in other laminopathies. Charcot-Marie-Tooth disease type 2, including the subtype caused by *LMNA* mutation, features axonal degeneration and the loss of large myelinated fibers, while decreases in nerve conduction velocity are mild or absent [87]. More *LMNA* mutations linked to peripheral nerve disorders were subsequently detected, most of which were truncating and located in the rod domain.

#### 3.4.5. Mandibuloacral Dysplasia: Bone and Adipose Tissue Involvement

In 2002, a link between mandibuloacral dysplasia (MAD) and *LMNA* mutations was demonstrated [88]. MAD is a rare autosomal recessive disorder. Affected individuals have an undersized jaw, underdeveloped clavicles, and exhibit other congenital bone abnormalities combined with partial lipodystrophy [89,90]. MAD-associated mutations are located in the same exons as lipodystrophy-causing variants, indicating that MAD is a more severe phenotype that affects bone development in addition to fat distribution. Mutations in *ZMPSTE24*, which encodes the lamin-A-processing enzyme, have also been associated with a similar phenotype, albeit more severe and with an earlier onset [91]. Consistent with the effects on protein localization predicted for these mutations, alterations observed in MAD patients include nuclear accumulation of prelamin A, dysmorphic nuclei, and chromatin disorganization, all of which become more severe with increasing age [92].

#### 3.4.6. Multiple Tissue Involvement (Progeroid Syndromes)

##### Hutchinson–Gilford Progeria Syndrome

Hutchinson–Gilford progeria syndrome is a rare disease characterized by rapid apparent aging, beginning in childhood [93,94]. Patients with this syndrome do not usually survive beyond the second decade of life, and typically die from myocardial infarction or stroke [95,96]. Notable features of this syndrome include growth impairment, sclerotic skin, prominent eyes, joint contractures, small jaw size, reduced levels of subcutaneous fat, hair loss, dimpling and mottling of the skin, fingertip tufting, and prominent cutaneous vasculature [96]. In 2003, Francis Collins and colleagues identified the gene responsible [97]. De novo splicing mutations, most located in exon 11 of *LMNA*, were found to underlie this severe disorder. These mutations result in deletion of the carboxyl terminus of prelamin A, giving rise to a truncated prelamin A variant that is not appropriately processed to lamin A. Other *LMNA* missense mutations located in the rod domain that result in abnormal RNA splicing within exon 11 have also been reported in variant progeroid syndromes [98,99,100]. Progeroid features are also observed in mandibuloacral dysplasia caused by *LMNA* mutations, as discussed above.

##### Malouf Syndrome

Two heterozygous missense mutations in the *LMNA* gene, A57P [98] and L59R [101,102], were found in 3 patients with dilated cardiomyopathy and hypergonadotropic hypogonadism [103]. Premature ovarian failure, dilated cardiomyopathy, lipodystrophy, and progressive facial and skeletal alterations (micrognathia and sloping shoulders, but not acroosteolysis) were observed in all three patients. Despite a progeroid-like appearance, none of the patients experienced severe growth failure, alopecia, or progressive atherosclerosis.

##### Restrictive Dermopathy

The most recently identified laminopathy is restrictive dermopathy (RD), which causes death shortly after birth and is characterized by intrauterine growth retardation, defects of the skin and skull, pulmonary hypoplasia, and microstomia. This syndrome is caused by recessive null *ZMPSTE24* mutations and, less commonly, dominant de novo *LMNA* mutations. The accumulation prelamin A (either truncated or normal-length) results in the formation of lamin A and lamin C aggregates and alterations in nuclear architecture [104,105].

#### 3.4.7. Combination of Phenotypes in Isolated Patients

Combinations of different phenotypes have also been reported. These include an individual who was a compound heterozygote for the R527H and V440M *LMNA* mutations, and showed some signs of mandibuloacral dysplasia as well as muscular involvement [106]. These two *LMNA* mutations could explain the patient’s two apparently unrelated phenotypes [107]; and that of a 65-year-old man with the novel nonsense mutation p.Q353X, who presented with amyotrophy of the lower limbs, arrhythmia and cardiac hypofunction, and neurological and electrophysiological alterations, suggesting spinal muscular atrophy type 3 [108].

#### 3.4.8. Mutational Architecture of *LMNA*

Some *LMNA*-related phenotypes (e.g., restrictive dermopathy, mandibuloacral dysplasia, Hutchinson–Gilford progeria syndrome (HGPS), and familial partial lipodystrophy) are associated with mutations located in specific sites of the gene. For other phenotypes, such as those involving skeletal and cardiac muscle, no specific mutation site within the protein has been identified. Various disease phenotypes are the result of the regulatory role of lamin A/C in a variety of cellular processes, including transcription, intracellular signaling, the maintenance of nuclear shape, nuclear pore spacing, and chromatin organization [109,110,111,112]. We suspect that the complex spectrum of these diseases is associated with developmental alterations that influence particular tissues, depending on the mutation type. These disorders include muscular, peripheral neurogenic, lipodystrophy, and premature aging syndromes, and are characterized by the involvement of tissues of mesenchymal origin.

### 3.5. Genes Involved in Glycemic Control (ABCC8, KCNJ11, GCK, HNF1A, HNF4A): Different Mutation Types (Loss vs. Gain-of-Function) Can Give Rise to Opposing Phenotypes, While Identical Mutations Can Give Rise to Opposing Phenotypes at Different Stages of Life

**Figure 8:**
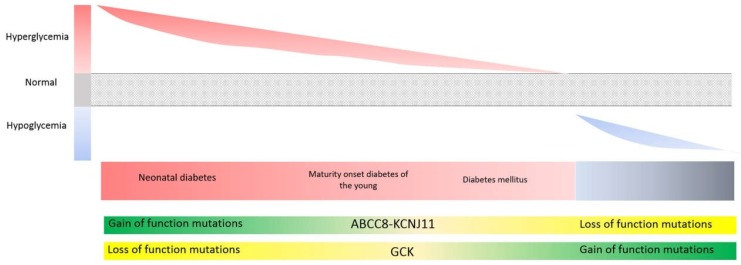
Phenotypic spectrum associated with mutations in genes involved in insulin secretion; *ABCC8*, *KCNJ11*, and *GCK*.

Here we will discuss a set of genes common to two completely opposing clinical entities: persistent hypoglycemia of infancy, and diabetes of varying severity (ranging from neonatal diabetes to non-insulin dependent diabetes mellitus) (Figure 8). Alterations in the genes *ABCC8*, *KCNJ11*, and *GCK* can result in phenotypes at opposite ends of the glycemic control spectrum (hypoglycemia or hyperglycemia), or alternatively a phenotype corresponding to the middle of the spectrum, resulting in maturity onset diabetes of the young (*MODY*), transient neonatal diabetes, or diabetes mellitus type II. These genes are involved different aspects of the regulation of glucose or insulin secretion. In the case of *HNF1A* and *HNF4A*, the same mutations give rise to hypoglycemia in the neonatal period and hyperglycemia in adulthood.

#### K_ATP_ Potassium Channel

The genes *ABCC8* and *KCNJ11* encode the two subunits of the ATP-dependent potassium channel (K_ATP_) of pancreatic β cells. This channel couples β-cell metabolism to membrane electrical events, regulating insulin secretion of β cells. Insulin secretion is stimulated by closure of K_ATP_ channels, and inhibited by channel opening. Loss-of-function mutations in either of these two genes result in either absence of these channels or in channel closure for longer than normal, causing a pernicious increase in insulin secretion and leading to states of persistent hypoglycemia that are very difficult to control. However, gain-of-function mutations result in opening of the channel for longer than normal, leading to decreased insulin secretion and hyperglycemia.

*GCK* encodes glucokinase, which catalyzes the first step of glycolysis and is expressed only in the mammalian liver and in the β cells of pancreatic islets. The activity of this enzyme determines the rate of glucose metabolism in the liver and pancreas. The higher the metabolic rate, the greater the increase in intracellular ATP and therefore the higher the level of insulin secretion (via the K_ATP_ channel). Decreases in enzyme activity result in corresponding decreases in intracellular ATP levels and insulin secretion. In 1992, loss-of-function variants in this gene were linked to the more frequent and less severe form of MODY-type diabetes [113]. Depending on the extent of loss of function and whether one or two of the alleles are affected, the resulting phenotype can range from severe diabetes that is present from birth to adult-type non-insulin-dependent diabetes mellitus [114,115,116]. A 1998 study [117] first described a gain-of-function variant in *GCK* in a patient with neonatal hypoglycemia associated with metabolic crisis and loss of consciousness. Other hypoglycemia-associated mutations were subsequently described.

Dominant inactivating mutations in *HNF1A* and *HNF4A* have long been associated with *MODY3* and *MODY1*. Recent studies have shown that the phenotype in individuals with these mutations is characterized by hyperinsulinism (HI) early in life that evolves to diabetes in later life [117,118,119]. Cases of diazoxide-responsive HI caused by mutations in *HNF1A* and *HNF4A* have been described, with resolution of HI during infancy or in childhood in some cases [120]. The clinical phenotypes were extremely variable. Two children showed evidence of ketone production during hypoglycemia, a biochemical profile atypical for hyperinsulinism. Given the heterogeneous clinical phenotypes of *HNF1A*-HI and *HNF4A*-HI, all children with transient, diazoxide-responsive HI without a clear history of perinatal stress should be screened for *HNF1A* and *HNF4A* mutations, as the results will help predict the clinical course and determine the subsequent management plan [120].

## 4. Inheritance

New mutations arise in heterozygotes and, depending on the strength of selection and the demographic history of the population in question, either increase in frequency or are rapidly eliminated [121,122,123,124,125]. Elucidating the relative contributions of the mutation type, natural selection, and genetic drift will help to explain why disease alleles persist in human populations. Answers to these questions are also of practical importance and facilitate the use of genetic variation data to identify additional disease mutations [126].

Knowledge of the mode of inheritance of variants is important when assessing their involvement in a patient’s clinical phenotype. In cases of dominant severe diseases, it is essential to determine whether the detected variant is an inherited or de novo mutation. Similarly, it is essential to determine whether variants detected in a recessive gene are located in opposite alleles before associating them with the disease.

Family-based WGS studies have shown that, on average, 74 germline SNVs occur de novo in an individual’s genome and one de novo mutation per exome [127]. The combination of WES with a patient-parent trio design allows detection of an average of 1.68 de novo mutations per patient, often enabling rapid identification of the gene underlying the patient’s condition [20].

De novo mutations are the most extreme form of rare genetic variation. In general, they are more deleterious than inherited variations; because they have been subjected to less stringent evolutionary selection [128,129]; these mutations are prime candidate causative genes underlying genetic diseases that occur sporadically.

Intolerant dominant genes are those that are most likely to be affected by de novo mutations. Any alterations in a mutation-intolerant gene result in such damage that it cannot perpetuate at the population level; the individual cannot pass it to their offspring because its effects prevent them from reproducing. Therefore, in the most conserved dominant genes, only de novo mutations are associated with pathologies. In fact, in the most extreme cases, only de novo mosaic mutations are allowed. This is because constitutive mutations in certain genes are incompatible with embryonic development, whereas in cases of mosaicism development can occur but is accompanied by very serious diseases, the etiology of which is hard to discern. Examples include McCune-Albright and Maffucci syndrome, and various chromosomal aneuploidy syndromes.

The prominent role of de novo mutations in rare diseases has been revealed in recent years thanks to the use of WES in trios [130]. Sequencing of parent-proband trios with ID [131,132], autism spectrum disorder (ASD) [133,134,135,136,137], schizophrenia (SCZ) [138,139], and epilepsy [21,140,141] suggests that de novo point mutations play an important role in pediatric and adult disorders of brain development.

## 5. Conclusions

To prioritize any variants detected by NGS analysis of patients with rare diseases, it is important

1. to first prioritize genes based on their tolerance to the mutation (*z*-score) and the a priori probability of finding one (for dominant) or two (for recessive) variants in this gene by chance (proportional to its length);
2. to characterize the mutational architecture of the gene in which the variants are located, i.e., how a given gene behaves in response to damage of different types (loss vs. gain-of-function mutations) or in different locations (e.g., protein domains);
3. to perform familial analyses after variant prioritization in order to determine the mode of inheritance of the variants and assess their pathogenicity.

By following these steps, the list of rare variants detected can be restricted to those most likely to be implicated in the clinical phenotype described, in some cases circumventing the need to perform functional studies.

## Figures and Tables

**Table 1:** List of the five most mutation-tolerant (>*z*-score) and mutation-intolerant (<*z*-score) of the 1670 genes associated with metabolic and neurological disorders for the four gene length ranges.

Most Intolerant Genes		Most Tolerant Genes	
Gene	*z*-Score	Gene	*z*-Score
<1000 bp		<1000 bp	
*COLEC11*	−1.377098	*TM4SF20*	2.215303
*CAV3*	−1.024795	*TMEM70*	2.215303
*CHCHD10*	−1.024795	*COQ4*	1.797098
*FAS*	−1.024795	*HACD1*	1.797098
*MRTO4*	−1.024795	*NDUFAF1*	1.41199
1000–2500 bp		1000–2500 bp	
*ACTG1*	−2.953587	*TSEN54*	3.541998
*CHRNA4*	−2.953587	*CHAT*	3.120652
*SDHA*	−2.889489	*WWOX*	2.700392
*ADCK3*	−2.566604	*TCN2*	2.667196
*BRF1*	−2.180352	*ASAH1*	2.634213
2500–5000 bp		2500–5000 bp	
*INSR*	−4.872409	*KIAA0556*	3.154369
*TRAPPC9*	−4.51124	*VWA3B*	2.667196
*GRIN2B*	−3.730224	*MOGS*	2.634213
*HECW2*	−2.953587	*CNTN2*	2.347576
*MAGI2*	−2.953587	*RECQL4*	2.347576
>5000 bp		>5000 bp	
*RYR1*	−10.002224	*TTN*	24.20914
*COL6A3*	−4.54985	*SYNE2*	8.331247
*FLNC*	−4.448544	*TNXB*	7.571299
*WDR81*	−4.025886	*ADGRV1*	6.627985
*PLEC*	−3.909057	*CENPF*	6.455138

**Table 2:** Dominant (**left**) and recessive (**right**) genes for which the probability of finding one or two variants, respectively, was the lowest for the four different gene length sections. Red, genes associated with metabolism; green, genes associated with epileptic encephalopathy.

Dominant Genes						Recessive Genes					
<1000 bp	*p*	1000–2500 bp	*p*	2500–5000 bp	*p*	<1000 bp	*p*	1000–2500 bp	*p*	2500–5000 bp	*p*
*HBA1*	0	*ACTB*	0	*ATP1A3*	0.0015	*ARL6IP1*	0	*ADK*	0	*HK1*	1.15 × 10^−6^
*HSPB3*	0	*BRAF*	0	*CNNM2*	0.0015	*ATP5E*	0	*ALG6*	0	*ZC3H14*	4.59 × 10^−6^
*NRAS*	0	*EEF1A2*	0	*EEF2*	0.0015	*BBIP1*	0	*CHST14*	0	*GRM1*	1.03 × 10^−5^
*PURA*	0	*GABRG2*	0	*KIF5C*	0.0015	*BOLA3*	0	*CLP1*	0	*ZAK*	1.03 × 10^−5^
*SDHD*	0	*GJC2*	0	*GRIN1*	0.0030	*C11ORF73*	0	*DNAJC3*	0	*DDHD1*	1.83 × 10^−5^
*SNAP25*	0	*GNB1*	0	*GRIN2D*	0.0030	*CLPP*	0	*ERLIN1*	0	*HACE1*	1.83 × 10^−5^
*STX1B*	0	*HSPD1*	0	*LZTR1*	0.0030	*COA5*	0	*GMPPB*	0	*MAGI2*	1.83 × 10^−5^
*THAP1*	0	*KIF2A*	0	*MYT1L*	0.0030	*COX14*	0	*MARS2*	0	*NDST1*	1.83 × 10^−5^
*VAMP1*	0	*LGI1*	0	*DNM1*	0.0045	*COX6B1*	0	*NEU1*	0	*OGDH*	1.83 × 10^−5^
*YWHAG*	0	*MAT1A*	0	*EFTUD2*	0.0045	*DNAJB2*	0	*NHLRC1*	0	*RAB3GAP1*	1.83 × 10^−5^
		*PPP3CA*	0	**>5000 bp**	***p***	*GM2A*	0	*POGLUT1*	0	***>5000 bp***	***p***
		*RAF1*	0	*CHD2*	0.0075	*MRPL44*	0	*SAMHD1*	0	*CAD*	4.11 × 10^−5^
		*TPM2*	0	*SCN2A*	0.0075	*NDUFA12*	0	*SLC39A14*	0	*CCDC88A*	5.60 × 10^−5^
		*TPM3*	0	*SCN1A*	0.0105	*PARK7*	0	*ST3GAL5*	0	*TRIO*	7.30 × 10^−5^
		*TREX1*	0	*SMARCA4*	0.0120	*PCBD1*	0	*TRMT10C*	0	*ARFGEF2*	1.37 × 10^−4^
		*TUBA1A*	0	*CACNA1B*	0.0135	*PCNA*	0	*WDR73*	0	*CC2D2A*	1.92 × 10^−4^
		*TUBB*	0	*SCN8A*	0.0135	*PDE6D*	0	*YME1L1*	0	*DOCK7*	1.92 × 10^−4^
		*TUBB2B*	0	*NALCN*	0.0150	*RPIA*	0			*LAMB2*	1.92 × 10^−4^
		*ZIC2*	0	*ARID1A*	0.0165	*TMEM138*	0			*ATR*	2.22 × 10^−4^
		*ZMYND11*	0	*DYNC1H1*	0.0165	*TXN2*	0			*CIT*	2.22 × 10^−4^
				*ITPR1*	0.0165	*UQCC3*	0			*ALS2*	2.90 × 10^−4^
